# Single Cell Genome Amplification Accelerates Identification of the Apratoxin Biosynthetic Pathway from a Complex Microbial Assemblage

**DOI:** 10.1371/journal.pone.0018565

**Published:** 2011-04-12

**Authors:** Rashel V. Grindberg, Thomas Ishoey, Dumitru Brinza, Eduardo Esquenazi, R. Cameron Coates, Wei-ting Liu, Lena Gerwick, Pieter C. Dorrestein, Pavel Pevzner, Roger Lasken, William H. Gerwick

**Affiliations:** 1 Center for Marine Biotechnology and Biomedicine, Scripps Institution of Oceanography, University of California San Diego, La Jolla, California, United States of America; 2 J. Craig Venter Institute, San Diego, California, United States of America; 3 Department of Computer Science and Engineering, Center for Algorithmic and Systems Biology, University of California San Diego, La Jolla, California, United States of America; 4 Skaggs School of Pharmacy and Pharmaceutical Sciences, University of California San Diego, La Jolla, California, United States of America; 5 Departments of Chemistry and Biochemistry, University of California San Diego, La Jolla, California, United States of America; Netherlands Institute of Ecology, The Netherlands

## Abstract

Filamentous marine cyanobacteria are extraordinarily rich sources of structurally novel, biomedically relevant natural products. To understand their biosynthetic origins as well as produce increased supplies and analog molecules, access to the clustered biosynthetic genes that encode for the assembly enzymes is necessary. Complicating these efforts is the universal presence of heterotrophic bacteria in the cell wall and sheath material of cyanobacteria obtained from the environment and those grown in uni-cyanobacterial culture. Moreover, the high similarity in genetic elements across disparate secondary metabolite biosynthetic pathways renders imprecise current gene cluster targeting strategies and contributes sequence complexity resulting in partial genome coverage. Thus, it was necessary to use a dual-method approach of single-cell genomic sequencing based on multiple displacement amplification (MDA) and metagenomic library screening. Here, we report the identification of the putative apratoxin. A biosynthetic gene cluster, a potent cancer cell cytotoxin with promise for medicinal applications. The roughly 58 kb biosynthetic gene cluster is composed of 12 open reading frames and has a type I modular mixed polyketide synthase/nonribosomal peptide synthetase (PKS/NRPS) organization and features loading and off-loading domain architecture never previously described. Moreover, this work represents the first successful isolation of a complete biosynthetic gene cluster from *Lyngbya bouillonii*, a tropical marine cyanobacterium renowned for its production of diverse bioactive secondary metabolites.

## Introduction

Understanding the mechanistic chemistry underlying the biosynthesis of bacterial polyketide and non-ribosomal peptide natural products has been greatly enhanced through access to the biosynthetic genes. For example, the DEBS gene cluster, encoding the production of the parent aglycone to the broad spectrum antibiotic erythromycin, provides the prototypical example [1]. Fortunately, in most cases, secondary metabolite biosynthetic genes are clustered in prokaryote genomes, a feature which facilitates their discovery and characterization [2], [3]. The current methods for locating biosynthetic gene clusters typically use homology-based hybridization or PCR screening of genomic libraries [4], or bioinformatic approaches with sequenced genomes [5], [6]. However, exotic and less well-studied organisms, such as tropical marine cyanobacteria, are found growing as complex microbial consortia, even when they are cultured in the laboratory, and genomic information is lacking. In these cases, the current gene targeting approaches are rendered inefficient or inappropriate (Fig. S4). Here, we expand upon previous genome screening approaches to aid in the identification of a putative biosynthetic gene cluster from a cultured sample of the marine cyanobacterium *Lyngbya bouillonii*. To this end, we strategically utilized recently developed methods that enable DNA sequencing from individual bacterial cells [7], [8] to facilitate subsequent location of a biosynthetic gene cluster in a metagenomic library. While single cell genome sequencing of prokaryotic organisms alone garners extensive information about gene cluster identification, >30% of the genome can be missing [9]. Further, the MDA amplification technique provides sufficient genomic DNA to enable ensuing biochemical investigation.

**Figure 1:**
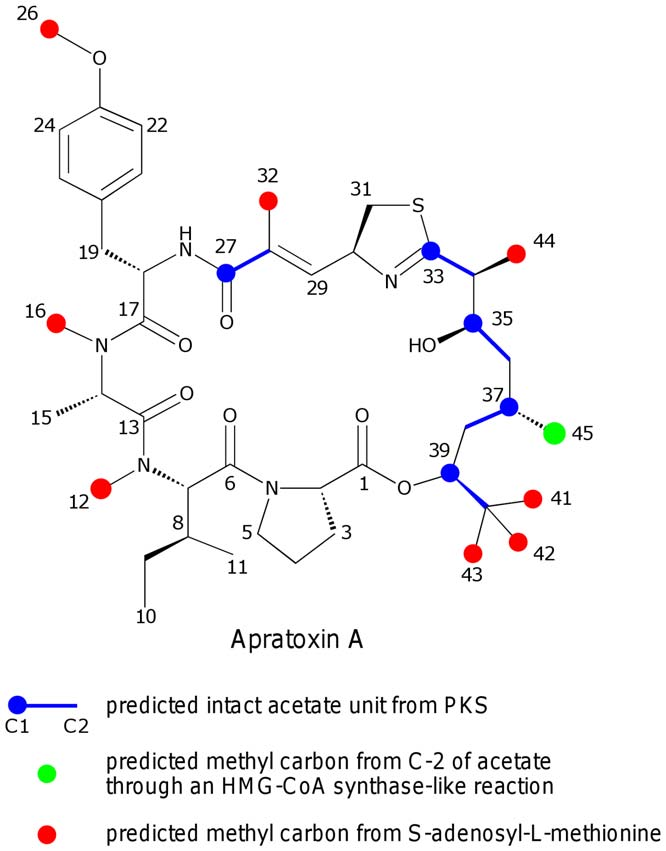
Predicted biosynthetic units of apratoxin A. Carbon atoms are numerically labeled.

Marine cyanobacteria have emerged as one of the most productive sources of highly bioactive and structurally novel natural product drug leads [10]. The spectrum of their biological activities is extremely broad, and notably includes antitubulin and antiactin agents, neurotoxins, and antibiotic substances [11], [12]. While a majority of these natural products derive from what has been described as a single species, *Lyngbya majuscula* [13], the Indo-Pacific species *L. bouillonii* is also a rich source of new natural products. To date, some thirty natural products spanning eight different chemical classes have been isolated from this latter species, thus revealing its exceptional biosynthetic capacity. Among these is the potent cancer cell cytotoxin, apratoxin A (**1**) (Fig. 1), which has been isolated from *L. bouillonii* inhabiting shallow coral reef environments surrounding the islands of Guam, Palau [14], [15] and Papua New Guinea [16]. Apratoxin A is a fascinating structure composed of a polyketide section fused with a modified pentapeptide to form a cyclic lipopeptide and possessing a unique tertiary butyl group at one terminus. Moreover, apratoxin A shows an extremely promising profile of selective cytotoxicity to cancer cells grown on solid agar media as well as *in vivo* anticancer effects in a mouse model [17]. Recently, the mechanism of cytotoxic action of apratoxin A has been described as involving the reversible inhibition of a secretory pathway for several cancer-associated receptors through interference with their co-translational translocation [18]. As a novel structure working by a new mechanism to potently and selectively kill cancer cells, it becomes of interest to understand its biosynthetic pathway and underlying genetic architecture, as well as the chemical mechanisms used in creating some of its distinctive structural features.

**Figure 2:**
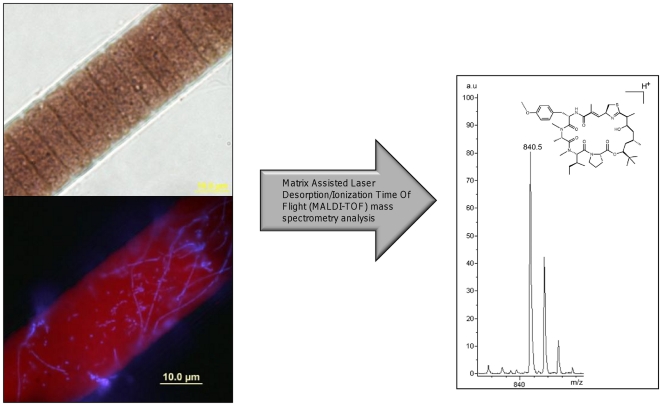
Microscopy and mass spectrometry of *Lyngbya bouillonii* filaments. (**a**) Light and fluorescent microscopy of a laboratory grown strain of *L. bouillonii* collected from Papua New Guinea. Brightfield (top) and 4′,6-diamidino-2-phenylindole (DAPI) stained and epi-fluorescent imaging (bottom) at 1000×. Cyanobacterial chlorophyll appears orange while filament sheath-associated bacterial DNA appears blue. (**b**) Matrix Assisted Laser Desorption Ionization-Time of Flight (MALDI-TOF) of an intact *L. bouillonii* filament demonstrates biosynthetic production of apratoxin A (obs. [M+H]^+^ *m/z* 840).

**Figure 3:**
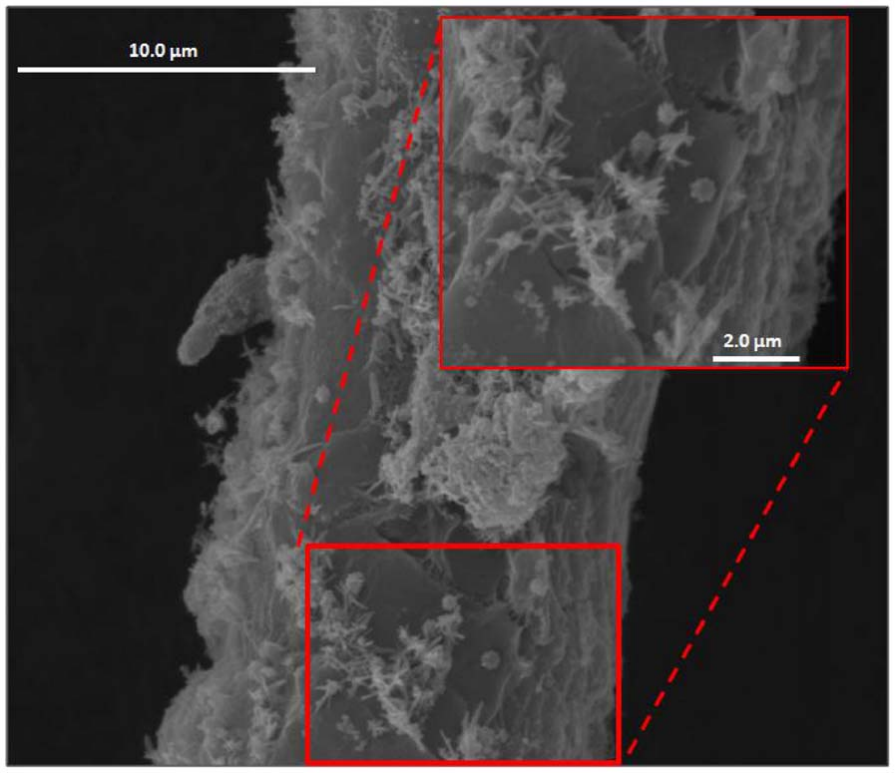
Scanning electron microscope image of a single *L. bouillonii* filament at 10,000× and 30,000× (inset) revealing heterotrophic bacterial growth on the sheath material of the cyanobacteria.

This filamentous cyanobacterium typically grows with a rich assortment of α-, β-, and γ-proteobacteria imbedded in and surrounding its thick polysaccharide sheath which encases the filament of cyanobacterial cells (Fig. 2a and Fig. 3) [19], [20]. Thus, it has not been possible to obtain axenic laboratory cultures of this species to date. However, we reasoned that this impediment might be overcome by isolation of single *L. bouillonii* cells from this microbial consortium and amplifying its genome by multiple displacement amplification (MDA) [21], [22], [23] to produce sufficient amounts of pure cyanobacterial DNA for partial genome sequencing. Although some of the genomic sequence is lost when attempting MDA from single cells, substantial amounts of the genome (60–70%) can be obtained [24]. MDA-generated DNA performs well as template in 454 pyrosequencing [25], and this method was chosen to obtain portions of the putative apratoxin biosynthetic gene cluster as identified through bioinformatic approaches. This sequence information was then used to design specific primers for the efficient identification of fosmids containing the apratoxin A pathway genes from a genomic library, ultimately leading to complete characterization of the gene cluster.

## Results

### Confirmation of apratoxin A production in cultured *L. bouillonii* by mass spectrometry

Uni-cyanobacterial cultures of the IndoPacific tropical marine cyanobacterium *Lyngbya bouillonii* were established by collection of portions of colonies using SCUBA and subsequent laboratory manipulations. Once acclimated to laboratory culture, this cyanobacterium grows robustly although with a relatively slow doubling time (approximately 12 days, Fig. 2a). Randomly selected filaments from a five liter culture were extracted with 2∶1 CH_2_Cl_2_/MeOH and the extract placed on a Matrix Assisted Laser Desorption Ionization (MALDI) plate. MALDI-time of flight (MALDI-TOF) analysis indicated that apratoxin A (**1**, *m/z* 840.5) was a major metabolite (Fig. 2b), and was accompanied by analogs with different levels of methylation (e.g. apratoxin B and C) [14], [26], [27] . To confirm that the *m/z* 840.5 Da ion corresponded to apratoxin A, this ion was subjected to fourier transform ion cyclotron resonance mass spectrometry (FT-ICRMS) and found to have a mass of 839.491 (theoretical mass *m/z* 839.487). A program specifically designed to annotate tandem mass spectra of cyclic peptides (MS-CPA) that contain non-standard subunits [28] was used to analyze fragments deriving from this ion, and confirmed that it was due to the presence of apratoxin A (Figs. S5 and S6).

Light and fluorescent microscopy with and without DNA staining with DAPI (4′,6-diamidino-2-phenylindole) revealed this *L. bouillonii* culture to be uni-cyanobacterial, but that the sheath material harbored a rich population of heterotrophic bacteria (Fig. 2a, inset). Recent comprehensive phylogenetic analysis of *Lyngbya*-associated microorganisms from laboratory cultured strains revealed that 44% (n = 95) of cloned 16S rRNA gene sequences belonged to non-*Lyngbya* taxa [29]. Unfortunately, even mild antibiotic treatment of these *L. bouillonii* cultures resulted in their rapid bleaching and death, and thus, these cultures are best characterized as microbial consortia. This result underscores the necessity of single cell genome sequencing approach to enhance sequence coverage of the *Lyngbya bouillonii* genome.

**Figure 4:**
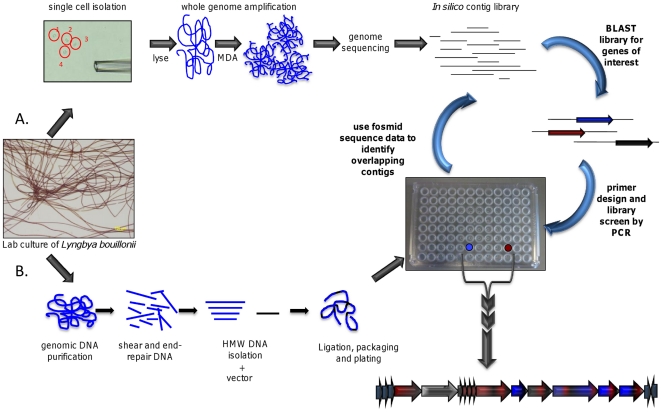
Consecutive strategies used to isolate the putative apratoxin biosynthetic gene cluster from a complex microbial assemblage. (**a**) Micromanipulation to isolate single cells used as template in multiple displacement amplification (MDA) and partial genome sequencing, and (**b**) metagenomic DNA from cultured non-axenic *L. bouillonii* used to create a fosmid library for subsequent PCR screening.

To obtain pure cyanobacterial DNA for genome sequencing, we isolated single cells from short filaments of *L. bouillonii* strain PNG/08/03/2001 using a micromanipulator and finely drawn glass micropipette made with a two-stage microcapillary puller [30]. Repetitive gentle pressure of the micropipette tip against the filaments dislodged single cells free of their surrounding sheath material. Four such single cells were sequentially recovered (Fig. 4), cycled through three washing steps with phosphate buffered saline (PBS) to exclude free DNA, and then placed separately into a lysis/reaction buffer mixture for DNA release and amplification.

The DNA of each of these four cells was subjected to whole genome amplification by MDA [22], a method of isothermal amplification that is based on the use of the bacteriophage φ29 DNA polymerase and random exonuclease-resistant primers [21]. The genome of each cell was rapidly amplified to yield approximately 5.0 µg of DNA. Consistent with earlier studies [7], this represents approximately a 10^9^ fold amplification from the few femtograms of DNA contained in a bacterial cell. To reconfirm that the amplified DNA was derived from *Lyngbya bouillonii* and that it was of reasonable quality for obtaining gene sequences, the 16S rRNA gene was PCR amplified from each MDA reaction and sequenced using the universal bacterial primers 27F/1492R [31], [32]. The 16S rRNA gene sequences from each amplified cell were greater than 99.6% identical. This result is within the expected range (0%–1.1%) of intragenomic sequence divergence between different 16S rRNA gene copies in *Lyngbya* single-cell MDA genomes [29]. Phylogenetic analyses showed that all sequences claded with *L. bouillonii* (Fig. S3). Moreover, BLAST analysis of the assembled sequences revealed *Lyngbya* as the only 16S rRNA gene sequence present in the *in silico* contig library (Dataset S1).

### Targeting the HMG-CoA synthase gene

A ß-alkylation event, wherein a branching carbon is attached to the C-1 carbon of an acetate subunit, is a distinctive biochemical transformation present in numerous of the polyketide secondary metabolites of cyanobacteria [33], [34]. This transformation requires the function of an HMG-CoA synthase-like (HCS) gene at the core of an extensive cassette of genes, typically embedded within a PKS pathway with a variety of optional genes providing variable tailoring to this pendant carbon atom. The genetic architecture and biochemistry of these variably modified ß-branching events have been reported for several natural products, including curacin A (AY652953) [4], jamaicamide A (AY522504) [2], mupirocin (AF318063) [35], bacillaene (U11039) [36], [37], pederin (AY059471) [38], and myxovirescin (NC_000964) [39]. Each of these clusters have stand alone homologs of a highly conserved HCS (70% to 98% identity at the amino acid level), as well as a set of genes encoding one or more ACPs, a mutant KS with a Cys-to-Ser active site substitution (KSs) and two homologs of the enoyl-CoA hydratase (ECH_1_ and ECH_2_) family. The enzymes encoded by these gene cassettes condense acetyl-CoA with the ß-ketoacyl-S-ACP intermediate of a growing polyketide chain. This results in the attachment of C-2 of the new acetate group, at various levels of oxidation and functionalization, to a C-1 position in the polyketide. Based on the structure of apratoxin A, we hypothesized that an HCS gene cassette was responsible for introduction of the C-45 secondary methyl group (Fig. 1). This highly conserved and distinctive gene motif was thus a convenient primary molecular marker for *in silico* contig screening for the apratoxin A biosynthetic gene cluster as well as subsequent metagenome library screening (Fig. 4).

Thus, before sequencing the MDA-generated DNA, we sought confirmation that this amplified DNA possessed at least one HCS homolog, as well as other signature NRPS/PKS biosynthetic genes. Thus, degenerate PCR primers were used to amplify the HCS-like gene [2] and general PKS primers were used to amplify the ketosynthase (KS) domains [40]. These primer sets were successful in amplifying approximately 650 bp and 700 bp fragments from the MDA template, respectively. The PCR products were sub-cloned and sequenced, and confirmed the presence of an HCS gene (∼86% identity to *jam*H (AAS98779) and *curD* (AAT70099) and several KS genes in the MDA genome (Fig. S2).

### Genome sequencing and assembly

The MDA amplified *L. bouillonii* DNA was sequenced using the following strategy. One half of a 454 FLX plate was allocated to a single cell MDA and the other half plate was a combination of four individually amplified cells. The rationale behind this strategy was to average potential coverage bias in the second half plate. Sets of reads were assembled into long contigs using a hybrid of 454 Newbler and EULER-SR assembly engines, as described below. Combining these two engines was necessary in order to recover sequences that were of low amplification or from regions difficult to sequence.

*De novo* assembly was first achieved using the 454 Newbler assembler. The resulting data consisted of 3,502 contigs >500 bp for a total coverage of 6.6 Mb. The largest contig was 26 kb while the average contig size was 2.2 kb. Initial screening of HCS signatures in the assembly generated by the 454 Newbler revealed that most belonged to short contigs (<2 kb), and these limited further analysis. However, as discussed below, one 2.05 kb contig (contig 04978) contained several of the domains recognizable in the HCS cassette (e.g. HMG, ECH_1_, ECH_2_,) and was instrumental to locating fosmids containing portions of the predicted apratoxin A cluster from a metagenomic library.

The Newbler engine was unable to produce longer contigs from the initial sequence data due to bias introduced during the amplification process and errors in the reads themselves. To overcome these limitations we explored methods to extend and merge the initially determined contigs. First, the EULER-SR assembler was used to recover sequence regions that were error prone or of low coverage. Moreover, this assembler corrects reads prior to assembly and enables a reduction in the ‘trustable coverage threshold’ for low coverage regions. The goal of this effort was to assemble as much novel sequence as possible which was not assembled by the 454 Newbler. Thus, we ran EULER-SR with parameters favorable for assembling low coverage regions. The assembly produced by EULER-SR resulted in about 5000 contigs that were larger than 500 bp with the largest contig being 12.5 kb.

**Figure 5:**
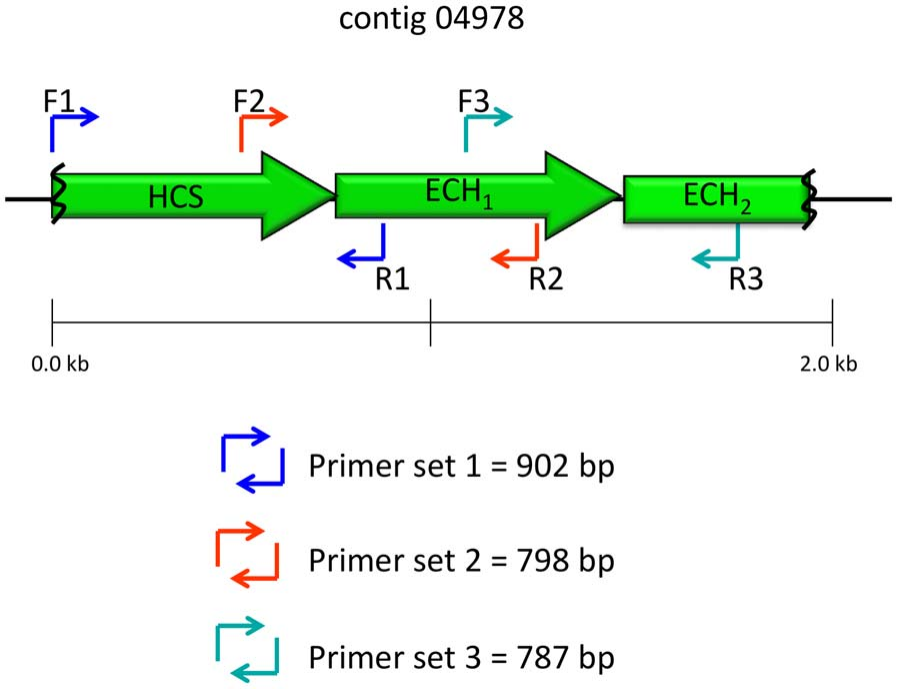
*In silico* determined contig, 04978, containing the distinctive HCS- ECH_1_-ECH_2_ catalytic motif [**2**]. Specific primers were designed (arrows) and used to PCR screen the metagenomic fosmid library. Domain nomenclature is the same as in Fig. 6.

Second, we extended and combined contigs generated by 454 Newbler with contigs produced by EULER-SR. Contigs were merged if they had a common seed larger than 30 bp and with a high alignment score. In total, 34 contigs between 10 kb and 42 kb were assembled, including one contig of 30 kb which had most of the signature HCS sequences. Ultimately, from the parallel fosmid sequencing efforts described below, this latter contig was mapped to the upstream and front half of the putative apratoxin biosynthetic gene cluster. All assembled and extended contigs >2 kb (2,164 contigs) were used in further analyses. In total, 6.5 Mb of unique sequence was obtained, yielding approximately 71–92% genome coverage based on the 7.1–9.1 Mb range of genome sizes for filamentous cyanobacteria [41]. This *in silico* contig library was screened by BLAST using an HCS consensus sequence so as to recover all such sequences in the library. This survey identified a single 2,050 bp contig (contig 04978) containing the recognizable HCS, ECH_1_, ECH_2_ domain architecture (Fig. 5). The laboratory production of apratoxin A combined with this finding gave confidence that the genome contained the biosynthetic cluster and that this *in silico* recovered HCS motif was likely part of the pathway.

### Isolation of the mixed PKS-, NRPS-, and HCS-containing locus from a metagenomic DNA library

The identification of a single contig containing the HCS-ECH_1_-ECH_2_ motif was essential for locating the putative apratoxin pathway from a metagenomic fosmid library (meta-gDNA, Fig. 4). The library was produced from high molecular weight (HMW) DNA obtained from laboratory cultured *L. bouillonii*. Approximately 1.0 µg of HMW-DNA was end repaired, ligated into a fosmid vector, packaged into phage particles and adsorbed into an engineered strain of *E. coli* (EPI 300) cells. A library of 1,642 colonies was obtained with an average insert size of 40–45 kb of genomic DNA in each fosmid. The colonies were arrayed into 96 well plates and screened by PCR using the ‘Piel pooling strategy’ [42].

**Figure 6:**
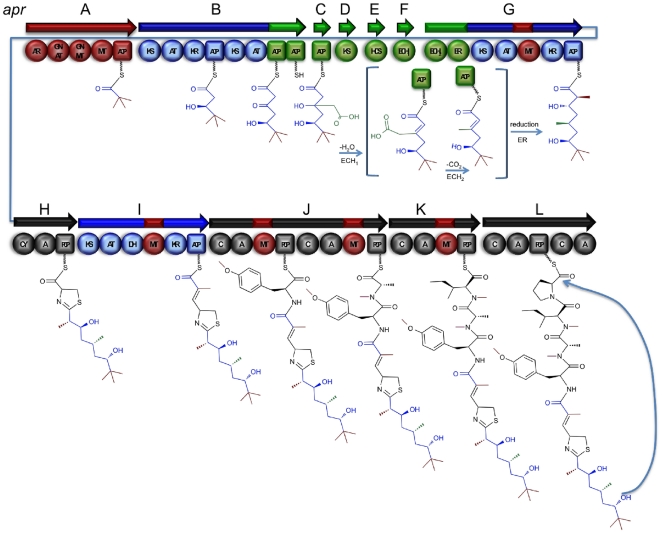
Proposed functions of the biosynthetic proteins produced by the *apr* pathway. Domain nomenclature is as follows: adapter region (AR), GCN_5_ acyltransferase (GNAT), glycine-N methyltransferase (GNMT), methyl transferase (MT), ketosynthase (KS), acyltransferase (AT), ketoreductase (KR), enoylreductase (ER), acyl carrier protein (ACP), HMG-CoA synthase (HCS), enoyl-CoA hydratase (ECH), condensation (C), adenylation (A), peptidyl carrier protein (PCP), cyclase (CY). The cluster has type I modular mixed polyketide synthase/non-ribosomal peptide synthetase (PKS/NRPS) organization containing 12 open reading frames, including a PKS-type loading module and nine extension modules.

Three sets of specific primers spanning various portions of the HMG, ECH_1_, and ECH_2_ motifs on contig 04978 were used to PCR-screen the metagenomic DNA library (Fig. 5). Six fosmids with varying degrees of overlap to each other were located, and end sequencing revealed that the DNA inserts harbored biosynthetic elements consistent with the predicted apratoxin biosynthetic pathway. Two fosmids calculated to provide greatest coverage of the pathway were selected for shotgun sequencing. Fosmid Apr1 contained a 38.8 kb insert which possessed 36.7 kb of the putative pathway as well as 2.1 kb of sequence upstream of the predicted loading module. Fosmid Apr2 was composed of 35.08 kb of insert DNA, all of which was deduced as part of the putative apratoxin pathway, extending from the HCS cassette domain through three PKS/NRPS extension modules and ending at an isoleucine condensation domain (*apr* K, Figs. 6 and S1). As neither fosmid provided complete coverage of the predicted cluster, a triple primer set PCR screen targeting both intra- and intergenic priming sequences from the downstream end of fosmid Apr2 was successful in identifying a third fosmid, Apr3, which contained additional sections of the putative apratoxin cluster. Unfortunately, this fosmid was chimeric, which likely occurred as a by-product formed during genomic library production from the ligation of two noncontiguous fragments. It also contained highly similar NRPS modules likely arising from genomic duplication events [43], and thus a very careful primer walking approach was taken to sequence key sections of this fosmid. In total, 21 rounds of primer design/gene walking were required to extend the cluster an additional 5.4 kb, and confirmation of part of this sequence was obtained from contig 03772 from the genome sequencing effort. Additionally, a second contig (00221) was found to overlap sections of the downstream end of fosmid Apr3, and extended the cluster by a further 3264 bp. Finally, a third PCR screen of the metagenomic library using the downstream sequence of contig 00221 revealed an overlapping fosmid, and this was also subjected to shotgun sequencing. This provided the remaining 4000 bp to the predicted terminus of the cluster, and a further 12.1 kb into DNA stretches which clearly did not encode for secondary metabolites (sugar transport proteins and transposases). To ensure that contig 00221 did in fact link fosmids Apr3 and Apr4, specific primers were used to PCR amplify this region from a genomic DNA prep produced from cultured *L. bouillonii*. Sequencing of the 3.5 kb amplicon confirmed the initial results, and thus, from data obtained from four separate contigs and partial or complete sequencing of four fosmids, the 57.4 kb sequence of the putative gene cluster for apratoxin A was obtained (Fig. 6).

### Gene Cluster Architecture and Proposed Biosynthesis

The 57,421 bp gene cluster is highly consistent with the predicted apratoxin A biosynthesis and will be referred to as the apratoxin (*apr*) pathway for brevity. The *apr* locus has type I modular mixed PKS/NRPS organization containing 12 open reading frames, including a PKS-type loading module and nine extension modules (four PKS and five NRPS) (Fig. 6). The pathway genes are flanked by putative transposases and coding regions for hypothetical proteins, providing provisional boundaries to the biosynthetic cluster.

The loading module for the apratoxin pathway contains regions of high identity (∼67%) to a family of GCN_5_-related transferases; methyltransferase (GNMT) and acyltransferase (GNAT) (Table 1). Thus, while several alternatives are conceivable, we predict the origin of the *t*-butyl terminus to derive from malonyl-CoA and three methyl groups donated from S-adenosyl-L-methionine (SAM). The homologous CurA GNAT (AAT70096) was recently shown to catalyze both the decarboxylation of malonyl-CoA to acetyl-CoA and to direct S-acetyltransfer from acetyl-CoA to load an adjacent ACP domain [44]. By analogy, the GNAT domain in the *apr* pathway could catalyze the decarboxylation of malonyl-CoA (**2**) and link this process to the function of the adjacent glycine *N*-methyltransferase (GNMT), a SAM (**3**) dependent methyltransferase known to methylate the nitrogen atom of glycine [45]. As in the case with the curacin A starter unit, the GNAT domain, in conjunction with its associated adaptor region (AR), is next predicted to transfer this mono-methylated substrate to the adjacent holo-ACP (**4**) where it likely undergoes two successive rounds of SAM-dependant methylation, catalyzed by the C-methyl transferase imbedded in this cassette of genes, to form compound **5** (Fig. S7).

**Table 1:** Deduced Functions of the Proteins in the *apr* Biosynthetic Gene Cluster.

protein	size (aa)	catalytic domains	proposed function	sequence similarity	identity/similarity
ORF1	90	transposase	transposase	*Cyanothece* sp. ATCC 51142	34%, 49%
ORF2	45	hypothetical protein	unknown	*Nostoc* sp. PCC7120	57%, 65%
ORF3	61	transposase	transposase	Lyngbya sp. PCC8106	77%, 86%
AprA	1139	AR, MT, AT, MT, T	2, 2,-dimethyl propionyl synthase	BryX, *Candidatus Endobugula sertula*	41%, 58%
AprB	2828	KS, AT, KR, T, KS, AT, T, T	PKS	CurI, *Lyngbya majuscula*	58%, 74%
AprC	84	T	acyl carrier protein	CurB, *Lyngbya majuscula*	79%, 89%
AprD	408	KS	keto-acyl synthase	JamG, *Lyngbya majuscula*	74%, 85%
AprE	418	HMGCS-like	Hmg-CoA sythase	JamH, *Lyngbya majuscula*	84%, 92%
AprF	259	ECH1	dehydration	JamI, *Lyngbya majuscula*	79%, 89%
AprG	2943	ECH2, ER, KS, AT, MT, KR, T	PKS	JamJ, *Lyngbya majuscula*	67%, 79%
AprH	1177	Cy, A_(cys)_, T	NRPS	CurF, *Lyngbya majuscula*	77%, 86%
AprI	1765	KS, AT, DH, MT, KR, T	PKS	JamJ, *Lyngbya majuscula*	58%, 73%
AprJ	2955	C, A_(Tyr)_, MT, T, C, A_(Ala)_, MT, T	NRPS	CrpC, *Nostoc* sp. ATCC 53789	56%, 71%
AprK	1264	C, A_(Ile)_, MT, T	NRPS	BarG, *Lyngbya majuscula*	58%, 73%
AprL	2126	C, A_(Pro)_, MT, T, C, A_(hydroxy-AA)_	NRPS, Heterocyclization	NosA, *Nostoc* sp. GSV224	45%, 61%
ORF4	77	hypothetical protein	unknown	N/A	N/A
ORF5	38	transposase	transposase	HctC, *Lyngbya majuscula*	84%, 89%

Gene functions were deduced using BLAST analysis. Abbreviations for catalytic domains are; A_(aa specificity)_; adenylation, AR; adapter region, AT; acyltransferase, C; condensation, Cy; cyclization, DH; dehydratase, ECH; enoyl CoA hydratase, ER; enoyl reductase, HMGCS; 3-hydroxy-3-methyl-glutaryl-CoA synthase, NRPS; non-ribosomal peptide synthase, PKS; polyketide synthase, KR; ketoreductase, KS; ketosynthase, MT; methyltransferase, T; thiolation.

AprB codes for two PKS extension modules with a tandem di-thiolation domain at the C terminus. While tandem acyl carrier protein organization is found in several PKS pathways [2], [4], [36], [39], [46], [47], relatively little is known about their mechanistic role in β-alkylation reactions. Deletion experiments of individual ACP domains in the mupirocin cluster revealed reduced activity for the pathway [46]. Consistent with this finding, a synergism in catalytic efficiency was observed in the range of β-branch processing steps from inclusion of the full complement of tandem ACP domains in the curacin A pathway [48]. In this latter work, evidence supported the role of these multiple ACPs in promoting a super-assembly of the biosynthetic enzymes. Nevertheless, the exact mechanisms by which they function are still largely unknown.

Following AprB is a series of genes homologous to an HCS-like gene cassette involved in the β-branching of polyketides, and includes the PCR targeted contig 04978. The deduced motif -ACP, KS, HCS, ECH_1_, ECH_2_, ER- ( = AprC, AprD, AprE, AprF, and the N terminus of AprG) is predicted to catalyze C-C bond formation between C-2 of an acetate group to the β-keto intermediate and reduce this to a pendant methyl group by dehydration, decarboxylation and double bond reduction. Next, the C-terminal region of AprG encodes for an acetate extension, C-2 methylation from SAM, and reduction of the β-keto carbon to a secondary alcohol. AprH has a predicted adenylation (A) domain specificity for cysteine, and optional domains for subsequent cyclization (Cy). By subdomain and adenylation specificity, the next five modules (one PKS and four NRPS) are predicted to successively add a C-methylated and unsaturated acetate unit followed by *O*-methyl tyrosine, *N*-methyl alanine, *N*-methyl isoleucine, and proline.

The final module of AprL is highly unusual. The condensation domain appears to be catalytically competent, whereas the functionality of the A-domain is uncertain. Primary sequence analysis indicates this domain could selectively activate amino acids with side chains possessing hydroxy groups. However, the universally conserved and active site residue lysine of the adenylation domain is absent, and this module also lacks an ACP. Moreover, a terminating thioesterase (TE) domain, which is required for release of the natural product, is conspicuously absent from the *apr* cluster. The canonical NRPS release mechanisms, including hydrolysis, macrolactam or macrolactone cyclization, or reduction, are known to occur when a chain terminating TE or Reductase (R) domain is present. However, cyclization and chain release activity of unique catalytic domains embedded in terminal NRPS modules has also been recently observed. For example the redox-incompetent R* domain in the cyclopiazinic acid biosynthetic pathway was reported to perform a Dieckmann-type condensation on an intermediate bound thioester and to release a cyclized product [49]. Further, the terminal NRPS portion of the FK520 biosynthetic gene cluster encodes a C1-A-PCP-C2 domain in which the proposed action of the second condensation domain (C2) is to putatively release the nascent compound by mediating macrolactonization via intramolecular nucleophilic attack of a hydroxy group onto the thioester carbonyl [50]. A third example is the final domain architecture of the mixed PKS/NRPS derived cyanobacterial compound, Aeruginoside in which a TE domain is absent [51]. The final two domains in the C-A-PCP-C-PCP module are proposed to be responsible for the catalytic release of the compound from the enzyme complex. Hence, in the absence of any other candidate proteins in the apratoxin A biosynthetic gene cluster, we speculate that the AprL condensation domain functions non-canonically to catalyze macrocyclization through a condensation-type mechanism.

## Discussion

Natural product chemical profiling of *L. bouillonii* over the past 20 years has yielded eight distinct classes of metabolites, consisting of nearly 40 different compounds [13]. Here, we report the first successful isolation of a complete biosynthetic gene cluster from this natural product rich organism. Identification of this cluster as that responsible for apratoxin biosynthesis is based upon a detailed bioinformatic deduction of precedented features as well as unique characteristics of the pathway, and their near perfect concordance with the expected sequence of enzyme activities based on the co-linearity rule [52]. Indeed, in all of the marine cyanobacterial NRPS/PKS secondary metabolite pathways so far sequenced, a very high degree of agreement with co-linearity has been observed [2], [3], [4], [40], [53]. Moreover, subsequent analysis revealed that only a single HMGCoA synthase-like domain (AprE) was present in the genome of this *L. bouillonii* strain, a feature required by the occurrence of a methyl group attached to a C-1-deriving position in the polyketide (e.g the C-37 methyl).

In the course of locating and characterizing the biosynthetic gene cluster for the anticancer natural product apratoxin A (**1**), we utilized new, single cell methodologies and a novel strategy to overcome some inherent problems with current approaches. Due to the close association of heterotrophic bacteria on the sheaths of filamentous cyanobacteria, it is not currently possible to obtain pure genomic DNA preparations from either field or laboratory cultured isolates. Thus, employing intact cyanobacterial filaments as a DNA source results in the formation of metagenomic DNA libraries. Additionally, the multitude of NRPS and PKS derived natural products in *L. bouillonii* are predicted to have highly similar genetic elements in their biosynthetic gene clusters, and hence, gene cluster targeting efforts are imprecise and can often result in the isolation of the incorrect pathway (Fig. S3). The utility of single cell isolation followed by whole genome amplification of only the cyanobacterial genomic DNA was both integral and necessary to the successful identification of the apratoxin A gene cluster. In part, this was made possible by the increasing access to low cost, high throughput DNA sequencing technologies that have appeared in recent years, such as 454 pyrosequencing [54] and Illumina technologies [55]. While the single cell-MDA approach does result in some genomic sequence being lost due to amplification bias or damage to the single genome copy, as much as 90% of the genome has been recovered through such approaches [56], [57].

The apratoxin A gene cluster is of particular interest due to the novelty of the chemical structure of the produced metabolite, the uniqueness of the biochemical reactions needed to produce several of its structural motifs, and its promising biological activity in cancer models. The loading domain architecture to form a *t*-butyl functionality is suggestive of a malonyl-CoA substrate which is decarboxylated and trimethylated from SAM, a surprising reaction sequence involving novel biosynthetic elements. While the genes encoding the enzymes for β-branch formation have been characterized in other cyanobacteria [33], [58], this is the first to observe those involved in the installation of a fully reduced methyl group (C-45). Finally, the offloading and cyclization of the initially formed linear apratoxin A scaffold represents a fundamentally new mechanism by which to effect these transformations. In composite, knowledge of the apratoxin gene cluster enables a detailed exploration of several new features of NRPS and PKS biochemistry as well as expands the range of structures and structural features accessed by these modular biosynthetic pathways.

Despite considerable gains over the past several years, the full promise of cyanobacterial natural products to yield new lead compounds and be developed as useful pharmaceuticals might only be realized after closing a series of key gaps in knowledge and technology. Addressing these challenges requires development and optimization of current genetic manipulation and genome sequencing of filamentous cyanobacteria. As reported here, we have overcome several major technical hurdles associated with MDA genome sequencing (incomplete genome coverage) and metagenomic library screening (contaminating heterotrophic DNA and chimeric fosmid sequences). Ultimately, to maximize sequence coverage and successfully locate the putative apratoxin A biosynthetic gene cluster, it was necessary for us to use both MDA-generated genomic information and traditional library screening methods. This dual-method approach can be adapted and applied to other mixed assemblages of organisms, particularly those that are difficult to sequence and/or are not readily amenable to current bioengineering methods (site directed mutagenesis, miRNA knockdowns, etc). In general, the use of MDA has enabled direct sequencing of environmental bacteria without the requirement for culture [7], [9], [59], [60], in essence accelerating access to pure genomes. Further, high throughput methods of single cell sorting (such as fluorescent activated cell sorting or FACS) are now routines and can be used upstream of the MDA reaction [24]. Indeed, innovative genomic approaches to natural products chemistry and chemical biology are contributing to a resurgence of interest in the application of natural products to biomedicine and drug discovery [61].

## Materials and Methods

### Routinely used bacterial strains and growth conditions available in Text S1

#### *Lyngbya bouillonii* Collection and Culture

Collection of *Lyngbya bouillonii* was made in Baru, Papua New Guinea (GPS location: 5°40.473 S and 146° 32.819 E) and given the collection number PNG/08/03/2001-10. Samples of the cyanobacterium were collected by hand at depths of 30–60 ft using SCUBA. In a field laboratory, filaments were separated and temporarily placed in growth vials containing local seawater. Following transfer to the culture laboratory, *L. bouillonii* was isolated from contaminating cyanobacteria and other microalgae using previously described techniques [62]. Laboratory cultures were maintained in SWBG11 in a controlled temperature room (28°C) with 16 h light/8 h dark cycles. Metagenomic DNA isolation and library construction methods are available in Text S2.

#### Intact cell MALDI-TOF MS. MALDI Matrix and sample preparation

One mL of MALDI matrix was composed of 70 mg of a mixture of 1∶1 α-cyano-hydroxycinnamic and dihydroxybenzoic acid (Universal MALDI matrix from Sigma), 750 µL acetonitrile, 248 µL milliQ H_2_0, and 2.0 µL of TFA. Using sterile tweezers, 3 or 4 filaments (roughly 5–10 µg wet weight) of *L. bouillonii* was placed in a 1.5 mL Eppendorf tube. About 1.0 µL of MALDI matrix solution per 1 µg biomass was added to the tube, and after 20–30 sec, 1.0 µL of this matrix extract was deposited in a well of a Bruker Microflex MSP 96 Stainless Steel Target Plate. After each spot had dried, the plate was placed in a Bruker Microflex MALDI-TOF equipped with flexControl 3.0. MALDI-TOF settings and detailed MS-CPA analysis available in Texts S3 and S4.

#### DAPI staining of *L. bouillonii*

Individual filaments of *L. bouillonii* were cultured under static conditions in fresh BG-11 for 30 days and mounted on glass slides using VECTASHIELD® Mounting Medium with DAPI (Vector Labs). The filaments were visualized on an Olympus IX51 using a DAPI/Hoeschst/AMCA Filter cube (Ex: 350/50, Em: 460/50). Images were captured using an Olympus DP70 camera.

#### Scanning electron microscopy (SEM)

*L. bouillonii* were cultured under static conditions in fresh BG-11 for 30 days and harvested for SEM preparation. Individual filaments were washed three times with phosphate buffer saline followed by three washes with sodium phosphate buffer. Dehydration of the samples was performed by soaking in 30%, 50%, 70%, 80%, 90% ethanol successively for ten minutes each condition and then for an hour in 100% ethanol. Critical point drying was performed followed by 60∶40 gold∶palladium sputter coating. Samples were visualized on an FEI Quanta 600 scanning electron microscope.

#### Single cell isolation and multiple displacement amplification

Filaments from *Lyngbya bouillonii* laboratory culture sample PNG/08/03/2001-10 were handled using sterile tweezers and lightly homogenized in sterile growth medium. The homogenate was kept on ice until micromanipulation. Individual cells from a single filament were isolated using mechanical micromanipulation as described previously [24]. The micromanipulator (TransferMan NK2; CellTram Vario, Eppendorf) was connected to an inverted microscope (Olympus IX70) and the sample was observed with a 40× objective. The separation of cells from the sheath was achieved using a capillary of approximate 20 µm inner diameter to first push neighboring cells out of the sheath followed by their capture and washing in sterile PBS-buffer. Finally, four cells were individually transferred to 0.5 µL lysis buffer (400 mM KOH and EDTA) in a 200 µL PCR tube (Eppendorf). Non-template control reactions were setup by transfer of approximately the same quantity of washing buffer to tubes containing only the lysis solution. The tubes were kept on ice until all cells were collected. The samples were incubated at 50°C for 10 min in a thermocycler (Eppendorf MasterCycler, Eppendorf). Whole genome amplification (Repli-g UltraFast kit, Qiagen) was carried out in a final volume of 5.0 µL by addition of 4.5 µL master mix to all tubes. The incubation time was 8 h at 30°C and the reaction was terminated by incubation at 65°C for 3 min (Eppendorf MasterCycler, Eppendorf). Quality of the amplified DNA was assessed by direct sequencing of the 16S rRNA PCR product using primers 27f (5′-TTA GAG TTT GAT CCT GGC TCA G-3′) and 1492r (5′-CGG TTA CCT TGT TAC GAC TT-3′).

#### Genome sequencing and assembly

Sequencing of an MDA reaction from one cell and also from a pool of four MDA reactions, each of which derived from a single cell taken from the same bacterial filament, was performed using 454 FLX pyrosequencing at the Joint Technology Center (JTC), J. Craig Venter Institute, Rockville, MD. Approximately 5.0 µg of the MDA product was used for 454 FLX library construction according to the recommended procedures of 454 Life Sciences.

The sequences from each one-half plate were assembled *de novo* using Newbler assembly software supplied by 454 Life Sciences. The dataset consisted of 567,000 unpaired reads generated by 454 FLX platform from DNA of the five individually amplified cells of *L. bouillonii* (one alone and four combined). All reads were longer than 50 bp and only 0.1% of reads were shorter than 100 bp; the average read length was 250 bp. Each read had a 4-nucleotide TAG prefix which was used to pool DNA, and about 80% of the reads had a large sequence (approx 30 nt) of primers.

The EULER-SR assembler was run with parameters favorable for assembling low coverage regions [63]. This assembly resulted about 5000 contigs (larger than 500 bp) with the largest contig 12.5 kb. Assembled and extended contigs >2 kb were used in further analyses.

#### Fosmid clone library screening

The *L. bouillonii* gDNA fosmid library, comprised of 1,632 colonies each containing approximately 40 kb of insert DNA, were arrayed in seventeen 96-well plates. Amplified fosmids from all 96 wells on each plate were pooled using a multiwell pipet (volume 100 µL/well), and diluted 1∶1 with LB broth. The matrix of seventeen 96-well plates was reduced to 17 pools representing the complete library. Each of these pools was used as template for PCR analysis as described below. Further, from each plate scoring positively from the primary PCR screen, pools were formed from each row (12 wells across) and each column (8 wells down) such that the 96 wells of one plate were reduced to 20 pools. The fosmid pools and mother plates (resealed with fresh sealing tape) were stored at −4°C.

#### Fosmid shotgun sequencing

Standard fosmid library construction and Sanger sequencing procedures [64] were completed at the J. Craig Venter Institute, Rockville, MD. Detailed bioinformatic analyses are provided in Text S5.

#### PCR cloning of HMG-CoA synthase and PKS gene homologs from the single cell MDAs

PCR amplification of probe fragments used in this study was performed with Taq DNA-polymerase (Promega) with the manufacturer's suggested concentration of template and primers in an Eppendorf Mastercycler gradient system. Conditions used were as follows: denaturation, 30 s at 94°C; annealing, 30 s at 48°C; extension, 60 s at 72°C; 30 cycles. Degenerate primers were designed based on two conserved sequences found in HCS-like cassette of genes and from PKS pathways (forward primer 5′- CTNCCNTAYGAYGAYCCCGT-3′ and reverse primer 5′-NCKRTGNGCNCCYTTNACCAT-3′). These primers were used to amplify a 650 bp fragment from *L. bouillonii* MDA-generated DNA. The amplicons were cloned into pGEM-T easy and sequenced. For amplification of the ketosynthase domain (KS) fragments from the *L. bouillonii* genomic DNA, previously designed primers KS1Up: 5′-MGI GAR GCI HWI SMI ATG GAY CCI CAR CAI MG-3′ KSD1: 5′-GGR TCI CCI ARI SWI GTI CCI GTI CCR TG-3′ were used. These primers amplified an approximately 700 bp fragment that was cloned into pGEM-T and sequenced.

## Supporting Information

Figure S1 **Sequencing map and gene arrangement of the 57.4 kb** ***apr*** **gene cluster from** ***L. bouillonii*** **.** Four contigs (light blue) and inserts of four fosmids (tan) containing the *apr* gene cluster are shown (size of molecules indicated in parentheses).
(TIF)

Figure S2 **Multiple sequence alignment of amplicons obtained from PCR-amplification of the HMGCoA synthase-like (HCS-like) domain from** ***L. bouillonii*** **metagenomic DNA.** Degenerate primers HCS forward (F1) and reverse (R1) were used to PCR amplify a 650 bp fragment from the purified metagenomic DNA. Subcloning and sequencing of 20 amplicons (HMGome clone) resulted in a single unique HCS sequence that was 100% identical to contig 04978.
(TIF)

Figure S3 **Evolutionary relationship of the 16S rRNA consensus sequence from the four single cell MDA amplified genomes (red arrow) to 7 taxa.** The evolutionary history was inferred using the Neighbor-Joining method [65]. The bootstrap consensus tree inferred from 1000 replicates is taken to represent the evolutionary history of the taxa analyzed [66]. Branches corresponding to partitions reproduced in less than 50% bootstrap replicates are collapsed. The percentage of replicate trees in which the associated taxa clustered together in the bootstrap test (1000 replicates) are shown next to the branches [66]. The tree is drawn to scale, with branch lengths in the same units as those of the evolutionary distances used to infer the phylogenetic tree. The evolutionary distances were computed using the Maximum Composite Likelihood method [67] and are in the units of the number of base substitutions per site. All positions containing alignment gaps and missing data were eliminated only in pairwise sequence comparisons (Pairwise deletion option). There were a total of 1987 positions in the final dataset. Phylogenetic analyses were conducted in MEGA4 [68].
(TIF)

Figure S4 **Partial gene architecture of another** ***Lyngbya bouillonii*** **-derived natural product biosynthetic gene cluster obtained through homology-based approaches.** Domain nomenclature is the same as Fig. 6. The type I modular polyketide synthase (PKS) system is comprised of a loading module, two extension modules, an HCS cassette, a third and fourth extension modules containing the full complement of reductive domains, and finally, an incomplete fifth module where only the ketosynthase (KS) and acyl transferase (AT) domains were sequenced. Thus, this partial gene cluster is inconsistent with the predictions for apratoxin biosynthesis and likely codes for the production of a similar, primarily PKS derived compound [69]. Domain nomenclature is the same as in Fig. 6.
(TIF)

Figure S5 **The observed apratoxin A fragmentation patterns when subjected to collision induced dissociation mass spectrometry (CID-MS).**
(TIF)

Figure S6 **Annotation of the Apratoxin MS/MS spectrum. Nomenclature forward by Ngoka and Gross was adopted** [**70**] **.**
(TIF)

Figure S7 **Proposed mechanism of formation of the** ***t*** **-butyl terminus of apratoxin A. Domain nomenclature is the same as in** **Fig. 6** **.**
(TIF)

Text S1 **Bacterial strains and growth conditions.**
(DOC)

Text S2 **Metagenomic DNA isolation and library construction.**
(DOC)

Text S3 **MALDI-TOF Settings.**
(DOC)

Text S4 **MS-CPA analysis.**
(DOC)

Text S5 **Bioinformatic analyses.**
(DOC)

Dataset S1 **BLAST analysis of the assembled and extended contig sequences (>2 kb) against the SILVA 16S rRNA database** [**71**] **revealed** ***Lyngbya*** **as the only 16S rRNA gene sequence present in the** ***in silico*** **contig library.**
(XLS)
