# Supplementary figures and images for: Single Cell Genome Amplification Accelerates Identification of the Apratoxin Biosynthetic Pathway from a Complex Microbial Assemblage
Source: PLoS One. 2011 Apr 12;6(4):e18565. doi: 10.1371/journal.pone.0018565 (PMC3075265; doi:10.1371/journal.pone.0018565)

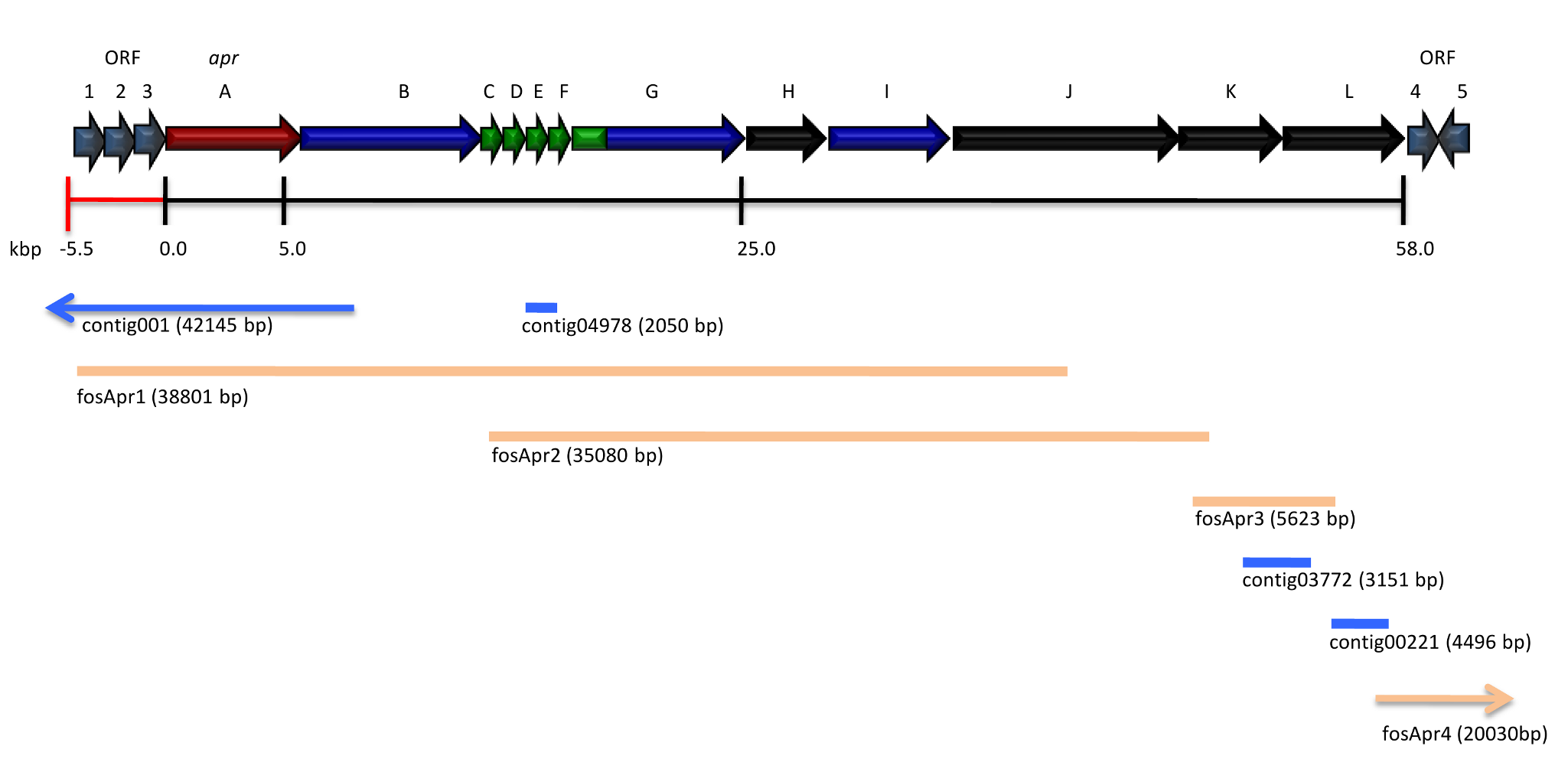

Supplement: Figure S1 — Sequencing map and gene arrangement of the 57.4 kb apr gene cluster from L. bouillonii . Four contigs (light blue) and inserts of four fosmids (tan) containing the apr gene cluster are shown (size of molecules indicated in parentheses). (TIF) [file pone.0018565.s001.tif]

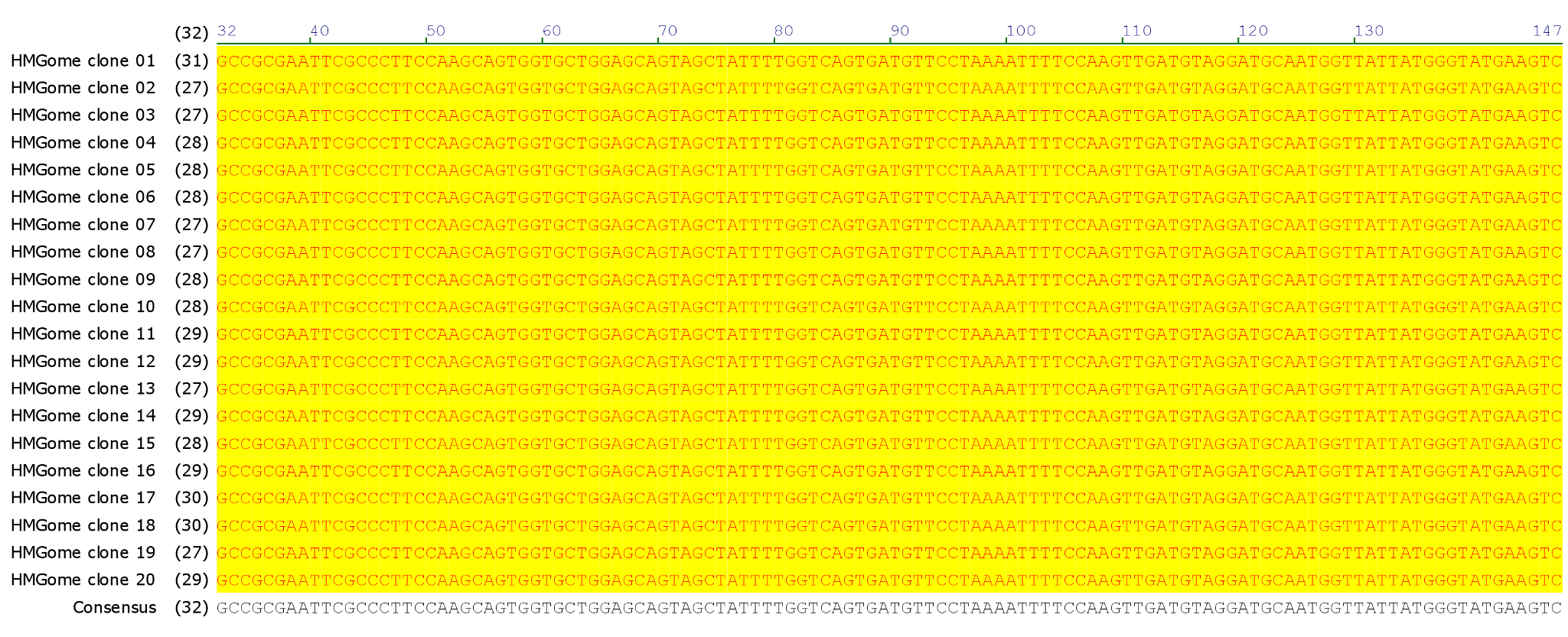

Supplement: Figure S2 — Multiple sequence alignment of amplicons obtained from PCR-amplification of the HMGCoA synthase-like (HCS-like) domain from L. bouillonii metagenomic DNA. Degenerate primers HCS forward (F1) and reverse (R1) were used to PCR amplify a 650 bp fragment from the purified metagenomic DNA. Subcloning and sequencing of 20 amplicons (HMGome clone) resulted in a single unique HCS sequence that was 100% identical to contig 04978. (TIF) [file pone.0018565.s002.tif]

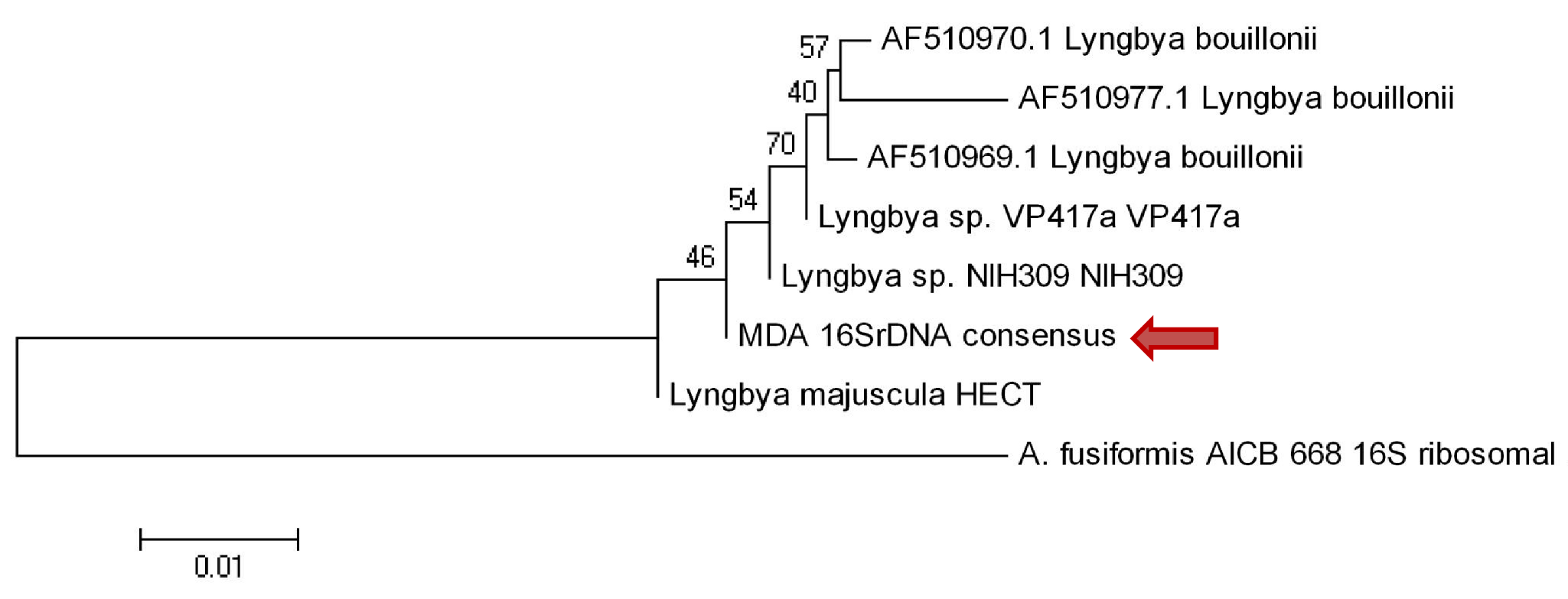

Supplement: Figure S3 — Evolutionary relationship of the 16S rRNA consensus sequence from the four single cell MDA amplified genomes (red arrow) to 7 taxa. The evolutionary history was inferred using the Neighbor-Joining method [65]. The bootstrap consensus tree inferred from 1000 replicates is taken to represent the evolutionary history of the taxa analyzed [66]. Branches corresponding to partitions reproduced in less than 50% bootstrap replicates are collapsed. The percentage of replicate trees in which the associated taxa clustered together in the bootstrap test (1000 replicates) are shown next to the branches [66]. The tree is drawn to scale, with branch lengths in the same units as those of the evolutionary distances used to infer the phylogenetic tree. The evolutionary distances were computed using the Maximum Composite Likelihood method [67] and are in the units of the number of base substitutions per site. All positions containing alignment gaps and missing data were eliminated only in pairwise sequence comparisons (Pairwise deletion option). There were a total of 1987 positions in the final dataset. Phylogenetic analyses were conducted in MEGA4 [68]. (TIF) [file pone.0018565.s003.tif]

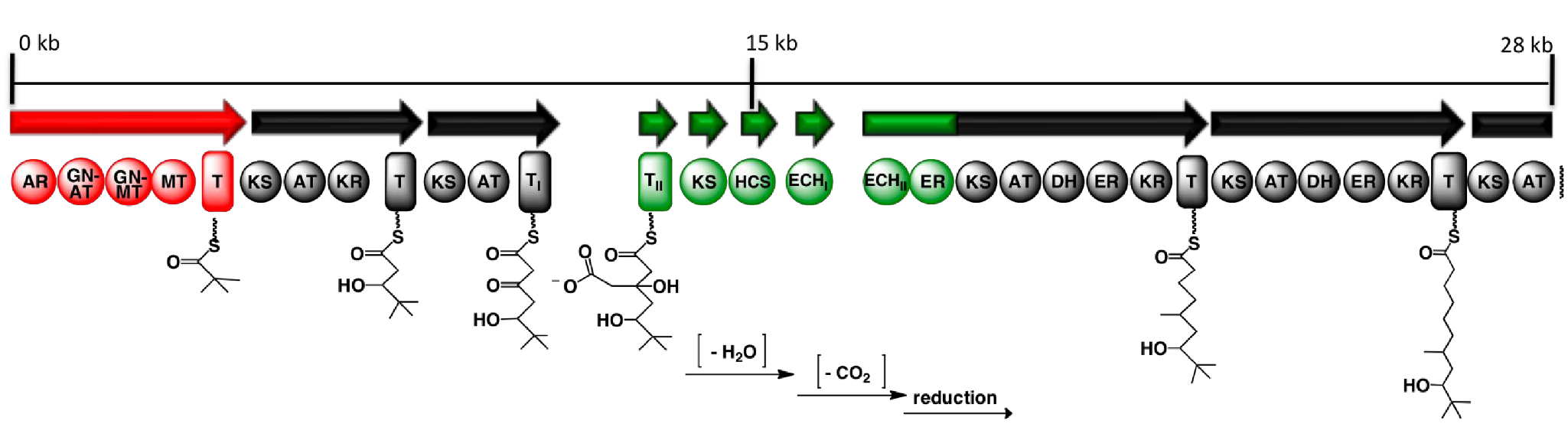

Supplement: Figure S4 — Partial gene architecture of another Lyngbya bouillonii -derived natural product biosynthetic gene cluster obtained through homology-based approaches. Domain nomenclature is the same as Fig. 6. The type I modular polyketide synthase (PKS) system is comprised of a loading module, two extension modules, an HCS cassette, a third and fourth extension modules containing the full complement of reductive domains, and finally, an incomplete fifth module where only the ketosynthase (KS) and acyl transferase (AT) domains were sequenced. Thus, this partial gene cluster is inconsistent with the predictions for apratoxin biosynthesis and likely codes for the production of a similar, primarily PKS derived compound [69]. Domain nomenclature is the same as in Fig. 6. (TIF) [file pone.0018565.s004.tif]

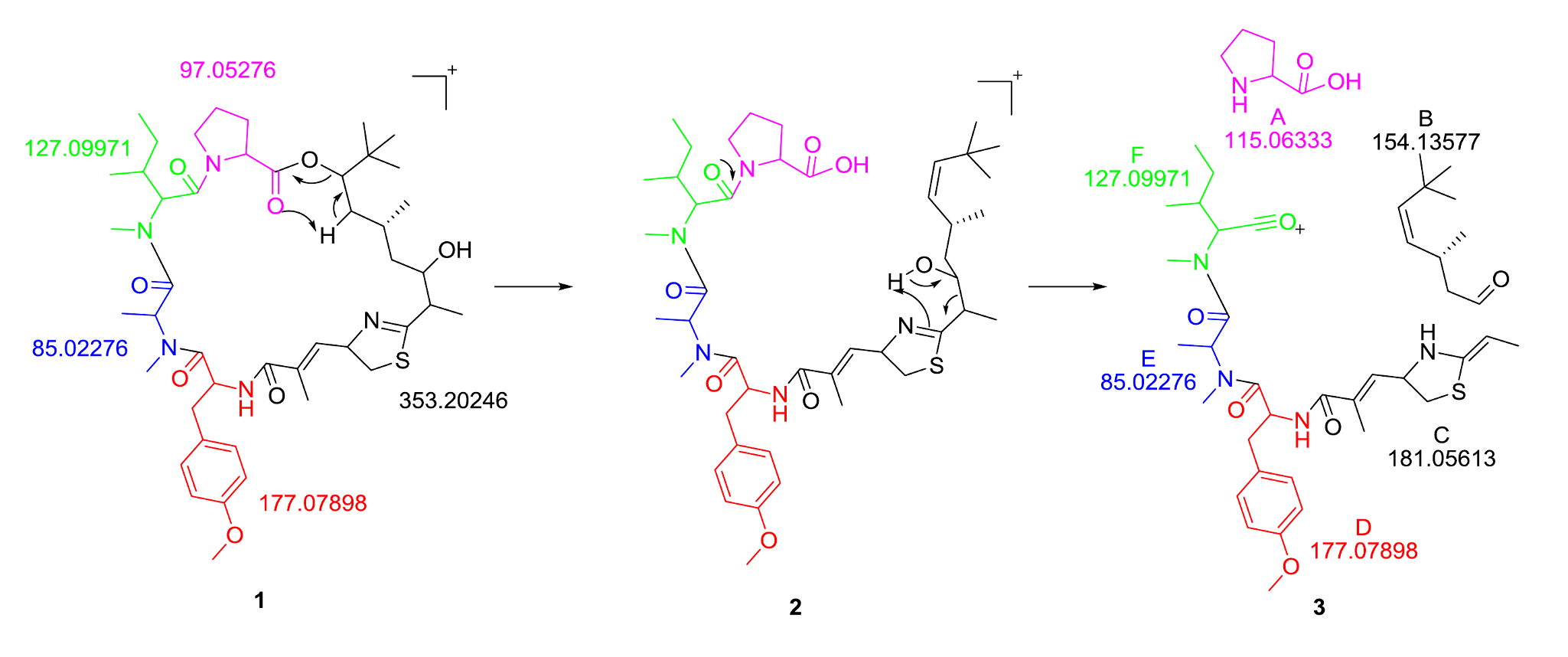

Supplement: Figure S5 — The observed apratoxin A fragmentation patterns when subjected to collision induced dissociation mass spectrometry (CID-MS). (TIF) [file pone.0018565.s005.tif]

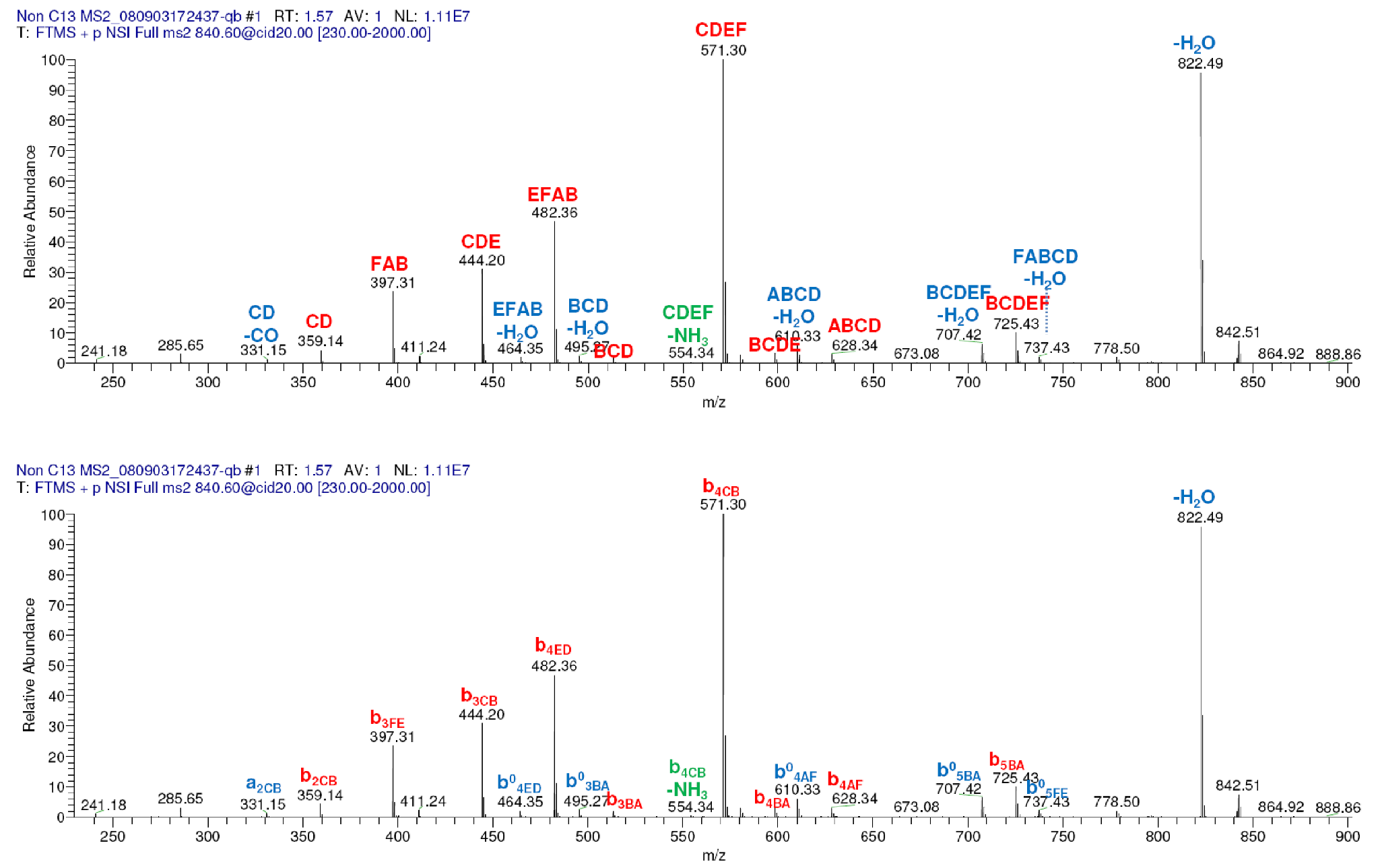

Supplement: Figure S6 — Annotation of the Apratoxin MS/MS spectrum. Nomenclature forward by Ngoka and Gross was adopted [70] . (TIF) [file pone.0018565.s006.tif]

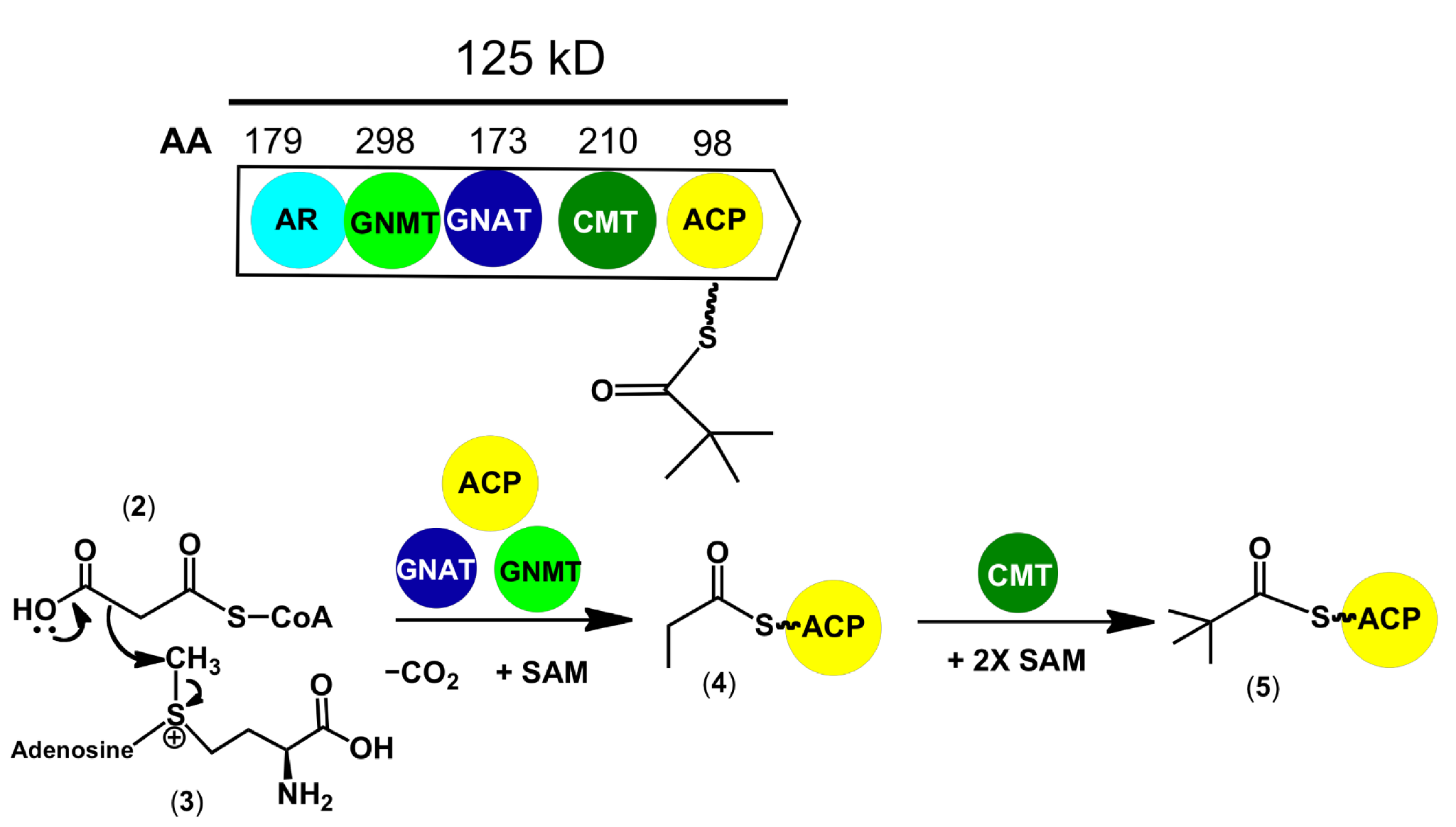

Supplement: Figure S7 — Proposed mechanism of formation of the t -butyl terminus of apratoxin A. Domain nomenclature is the same as in Fig. 6 . (TIF) [file pone.0018565.s007.tif]
