# Supplementary material for: Single Cell Genome Amplification Accelerates Identification of the Apratoxin Biosynthetic Pathway from a Complex Microbial Assemblage
Source: PLoS One. 2011 Apr 12;6(4):e18565. doi: 10.1371/journal.pone.0018565 (PMC3075265; doi:10.1371/journal.pone.0018565)
Supplement: Text S1 — Bacterial strains and growth conditions. (DOC) [file pone.0018565.s008.doc]

**Text S1 Bacterial strains and growth conditions.** *Escherichia coli* strain EPI300 was used in this study as a host for fosmid library construction and amplification of fosmid clones. One Shot® TOP10 chemically competent *E. coli* containing pCR2.1-TOPO or pGEM-T easy was grown overnight in LB (Luria-Bertani) medium with ampicillin at a final concentration of 100 mg/mL. The EPI300 strain of *E. coli* harboring fosmid vector was grown overnight in LB medium with chloramphenicol at a final concentration of 12.0 mg/mL.
