# Supplementary material for: Single Cell Genome Amplification Accelerates Identification of the Apratoxin Biosynthetic Pathway from a Complex Microbial Assemblage
Source: PLoS One. 2011 Apr 12;6(4):e18565. doi: 10.1371/journal.pone.0018565 (PMC3075265; doi:10.1371/journal.pone.0018565)
Supplement: Text S2 — Metagenomic DNA isolation and library construction. (DOC) [file pone.0018565.s009.doc]

**Text S2 Metagenomic DNA isolation and library construction.** DNA from laboratory cultured *L. bouillonii* PNG/08/03/2001-10 was used for PCR amplification. The Wizard Kit (Promega, Madison, WI) was used to isolate genomic DNA according to manufacturer suggestions. High molecular weight (HMW) DNA from cultured PNG/08/03/2001-10 was used for library construction, as previously described . The HMW-DNA was end repaired, size selected, and ligated into the copy control fosmid vector pCC1FOS using protocols provided with CopyControl^TM^ Fosmid Library Production Kit (Epicentre Biotechnologies, Madison, WI). Plasmid preparations were carried out using commercial kits (Qiagen, Santa Clarita, CA) and (FosmidMAX™ Epicentre Biotechnologies, Madison, WI). Other basic DNA manipulations, such as restriction digests and ligations, were performed using standard methods.
