# Supplementary material for: Single Cell Genome Amplification Accelerates Identification of the Apratoxin Biosynthetic Pathway from a Complex Microbial Assemblage
Source: PLoS One. 2011 Apr 12;6(4):e18565. doi: 10.1371/journal.pone.0018565 (PMC3075265; doi:10.1371/journal.pone.0018565)
Supplement: Text S3 — MALDI-TOF Settings. (DOC) [file pone.0018565.s010.doc]

**Text S3 MALDI-TOF Settings*.*** The instrument and program settings for these experiments were as follow: *General*: Flex-Control Method- RP_pepmix.par. *Processing*: Flexanalysis Method- none, Biotools MS method- none. Laser Power: 30-38 % Sample Carrier: none. Spectrometer: On, Ion Source 1- 19.0 0 mV, Ion Source 2- 16.40 mV, Lens- 9.45 mV, Reflector 20.00, Pulsed Ion Extraction- 190 ns, Polarity- Positive. Matrix Suppression: Deflection, Suppress up to: *m/z* 500. Detection: Mass Range- 500-1600, Detector Gain- Reflector 3.7X. Sample Rate- 2.00 GS/s, Mode- low range, Electronic Gain-Enhanced, 100 mV. Real time Smooth- Off. Spectrometer, Size: 81040, Delay 42968. Processing Method: Factory method RP_2465. Setup: Mass Range- Low. Laser Frequency- 20 Hz, Autoteaching- off. Instrument Specific Settings: Digitizer- Trigger Level- 2000 mV, Digital Off Linear- 127 cnt, Digital Off, Reflector- 127 cnt. Detector Gain Voltage Offset, Linear- 1300 V, Reflector- 1400 V. Laser Attenuator, Offset -12 %, Range- 30 %, Electronic Gain Button Definitions, Regular: 100 mv (offset lin) 100 mV (offset ref) 200mV/full scale. Enh: 51 mV (offset lin), 51 mV (offset ref) 100 mV/full scale. Highest: 25 mV (offsetlin) 25 mV (offset ref) 50 mV/full scale. Calibration: Calibration was accomplished using angiotensin II as an external standard. Zoom Range +-1.0%, Peak Assignment Tolerance- User Defined-500 ppm.
