# Supplementary material for: Single Cell Genome Amplification Accelerates Identification of the Apratoxin Biosynthetic Pathway from a Complex Microbial Assemblage
Source: PLoS One. 2011 Apr 12;6(4):e18565. doi: 10.1371/journal.pone.0018565 (PMC3075265; doi:10.1371/journal.pone.0018565)
Supplement: Text S4 — MS-CPA analysis. (DOC) [file pone.0018565.s011.doc]

**Text S4 MS-CPA analysis.** The 840.491 Da ion was isolated in the mass spectrometer and fragmented by collision induced dissociation and the resulting MS/MS spectrum was collected. The resulting spectrum was subjected to MS-CPA analysis. MS-CPA was used to aid the analysis of the tandem mass spectrometric data . Initially we tried to annotate the tandem mass spectrum to the apratoxin A sequence tags 97-353-177-85-127 (subunits showed in Fig. S4, structure 1), but less than 5% of the spectral intensity could be annotated. Since apratoxin has a lactone ring in its structure, and scrutinizing the spectrum, a strong tendency to lose 154 Da was observed. Both of these observations suggested that apratoxin undergoes two rearragements for which the end products resemble McLafferty type rearrangements. Both rearrangement routes, for both the lactone as well as the rearrangement with the thiazole-hydroxyl group (Fig. S4). When these two rearrangements we accounted for, a new tag was generated as 115-154-181-177-85-127. Using this sequence tag, MS-CPA was able to annotate 82.75% of the intensity found in the tandem MS data confirming that this ion is indeed apratoxin A (Fig. S5). These results can be viewed at <http://lol.ucsd.edu/cyc_results_v1/71F8B9B9-C920-466A-8B37-D880B1B8DE82/annotations/index.html>
