# Supplementary material for: Single Cell Genome Amplification Accelerates Identification of the Apratoxin Biosynthetic Pathway from a Complex Microbial Assemblage
Source: PLoS One. 2011 Apr 12;6(4):e18565. doi: 10.1371/journal.pone.0018565 (PMC3075265; doi:10.1371/journal.pone.0018565)
Supplement: Text S5 — Bioinformatic analyses. (DOC) [file pone.0018565.s012.doc]

**Text S5 Bioinformatic analyses.** The assembled and extended 454 sequence contigs were uploaded to the personal sequence database (PSD) hosted by Oregon State University . Multiple database and sequence analysis functions, including database searching by keyword and BLAST sequence, similarity searching, protein domain identification, and multiple sequence alignments were performed via the PSD for initial contig and domain identification. Vector NTI® 10.3 (Invitrogen) and CLC bio workbench was used for routine DNA and protein sequence analysis. Database searches for genes and proteins for sequence homology with the apratoxin genes and putative polypeptides were performed by using the NCBI (National Center for Biotechnology Information) BLAST server (http://www.ncbi.nlm.nih.gov/), and Pfam (Protein Families Database (<http://pfam.sanger.ac.uk/>).
